# *Acidobacteria* strains from subdivision 1 act as plant growth-promoting bacteria

**DOI:** 10.1007/s00203-016-1260-2

**Published:** 2016-06-23

**Authors:** Anna M. Kielak, Matheus A. P. Cipriano, Eiko E. Kuramae

**Affiliations:** Department of Microbial Ecology, Netherlands Institute of Ecology (NIOO-KNAW), P.O. Box 50, Droevendaalsesteeg 10, 6708 PB Wageningen, The Netherlands

**Keywords:** *Acidobacteria*, PGPB, IAA, Root biomass, *Arabidopsis thaliana*

## Abstract

*Acidobacteria* is one of the most abundant phyla in soils and has been detected in rhizosphere mainly based on cultivation-independent approaches such as 16S rRNA gene survey. Although putative interaction of *Acidobacteria* with plants was suggested, so far no plant–bacterial interactions were shown. Therefore, we performed several in vitro tests to evaluate *Acidobacteria*–plant interactions and the possible mechanisms involved in such interaction. We observed that *Arabidopsis thaliana* inoculated with three strains belonging to *Acidobacteria* subdivision 1 showed increase in biomass of roots and shoots as well as morphological changes in root system. Our results indicate that the plant hormone indole-3-acetic acid production and iron acquisition are plausibly involved in the plant and *Acidobacteria* interactions. Here, we confirm for the first time that *Acidobacteria* can actively interact with plants and act as plant growth-promoting bacteria. In addition, we show that *Acidobacteria* strains produce exopolysaccharide which supports the adhesion of bacteria to the root surfaces.

## Introduction

*Acidobacteria* is a very diverse and ubiquitous bacterial phylum. Furthermore, those bacteria seem to be especially well adapted to soil environment, often representing one the most abundant bacterial phylum (Janssen 2006; Lee et al. 2008). Although there are inconsistencies in reports regarding the preference of *Acidobacteria* in inhabiting bulk versus rhizosphere soils (Fierer et al. 2007; Singh et al. 2007; Kielak et al. 2008), there are clear evidences for the association of some *Acidobacteria* with plants (da Rocha et al. 2010, 2013). The enormous phylogenetic diversity within the phylum also suggests that *Acidobacteria* are genetically and, most likely, metabolically dissimilar; thus, the results of single studies cannot be generalized and easily extrapolated to the whole phylum.

Due to the still low number of sequenced genomes and difficulties associated with cultivation, the ecological role of this phylum remains rather unknown (Kielak et al. 2016). Nevertheless, a number of studies have compared distribution and diversity of *Acidobacteria* in relation to plant root proximity (Chow et al. 2002; Filion et al. 2004; da Rocha et al. 2010; Chaparro et al. 2014) and/or plant exudates (Shi et al. 2011; Mao et al. 2014). For example, acidobacterial strains have been obtained from internal plant tissues hinting to an endophytic lifestyle (Idris et al. 2004; Nissinen et al. 2012; Poosakkannu et al. 2015). Mendes et al. (2014) using culture-independent approach technique have shown that *Acidobacteria* are overrepresented in soybean rhizosphere, and da Rocha et al. (2010) have reported by means of qPCR, the *Holophagae* (*Acidobacteria* subdivision 8) being more abundant in leek rhizosphere. However, in the second case bacterial cell number was lower in spheres very proximate to roots or on the root surface itself.

*Acidobacteria* were also shown to be dominant in the rhizosphere of *Arabidopsis thaliana*, and moreover, change in terms of phylum composition and abundance during plant development, possibly due to changes in plant exudation (Chaparro et al. 2014). However, most of the studies were based on culture-independent method based on 16S ribosomal gene marker sequencing due to difficulty to culture *Acidobacteria* and perform experiments under laboratory conditions. Thus, those type of studies do not investigate the nature of plant–bacteria interactions. Concerning few studies on *Acidobacteria* physiology, non-traditional sources of carbon such as complex polysaccharides were suggested to improve cultivability of *Acidobacteria* (Koch et al. 2008; Pankratov and Dedysh 2010; Eichorst et al. 2011). Also some of the characterized strains were shown to be able to utilize plant-derived polymers (Pankratov et al. 2008, 2012; Eichorst et al. 2011) further suggesting close relation between plants and specific *Acidobacteria* subdivisions. Nevertheless, available strains can be used for attempts of studying interactions with plants under experimental conditions. In order to test the hypothesis that *Acidobacteria* strains effect plant growth, we assayed the interactions of three class *Acidobacteria* strains with *A. thaliana* ecotype Columbia 0 (Col 0) under in vitro conditions.

## Materials and methods

### Bacterial strains

Three *Acidobacteria* strains belonging to the class *Acidobacteria* from the NIOO-KNAW microorganisms’ collection were used in this study. Two of the strains are affiliated with genus *Granulicella*, namely *Granulicella* sp. WH15 (Valášková et al. 2009) and 5B5 (KM979383), and one is a type strain of the genus *Acidicapsa*, *A. ligni* WH120T (Valášková et al. 2009; Kulichevskaya et al. 2012). *Pseudomonas putida* IAC-RBal4 (KJ590499) and *Escherichia coli* WA321 (DSM no. 4509) strains were used as positive and negative controls of plant growth-promoting bacteria, respectively.

### Plant–bacteria interaction experiment

*Arabidopsis* *thaliana* ecotype Columbia 0 seeds were surface sterilized by washing in 70 % ethanol for 5 min followed by submerging for 10 min in 50 % bleach and rinsing four times with sterile distilled water. Sterile seeds were placed on half-strength Murashige and Skoog (MS) medium pH 5.7 (Murashige and Skoog 1962) supplemented with 12 g L^−1^ plant agar (Duchefa Biochemie bv) and 5 g L^−1^ sucrose. Six plants were grown per plate. Seedlings were incubated at 21 °C with the light cycle of photoperiod 16 h/8 h day/night. The root tips of 5-day-old seedlings were inoculated with 2.5 µL of bacterial suspension (phosphate saline buffer, pH 5.5) of OD600 = 1 corresponding to 1.7 × 10^6^, 1.5 × 10^7^ and 1.3 × 10^7^ CFU for *A. ligni* WH120T, *Granulicella* sp. 5B5 and WH15, respectively, or by direct transfer from the solid medium (0.1 × TSA, pH 5.0 see below) as an alternative method. The effect of bacteria on plant growth was evaluated 3 weeks post-inoculation.

### Indole acetic acid (IAA) production

IAA production was determined based on the method described by Bric et al. (1991). *Acidobacteria* strains and *P. putida* IAC-RBal4 (positive control) were inoculated on 0.1 × tryptone soy agar (TSA) plates (pH 5.0 and 5.7) supplemented with 5 mM L^−1^ tryptophan and covered with a cellulose nitrate filter (0.45 µm pore size, Sartorius). TSA contained 1 g L^−1^ NaCl, 3.0 g L^−1^ TSB (Oxoid), 1.95 g L^−1^ MES, 20 g L^−1^ agar (Boom, Netherlands). Plates were incubated at 20 °C until colonies reached approximately 4–5 mm diameter (5 days), and then the membranes were washed in the Salkowski reagent (1.2 % FeCl_2_ in 37 % sulfuric acid). The reaction was allowed to proceed for 30 min at RT until purple color appeared. All strains were tested in triplicates on separate plates.

### Phosphate solubilization assay

*Acidobacteria* strains were tested for their ability to solubilize a mineral form of phosphate. *P. putida* IAC-RBal4 was used as a positive control. Tests were performed on the National Botanical Research Institute’s phosphate growth medium (NBRIPM) containing per liter 15 g agar–agar ultrapure (Merck KGaA), 10 g glucose, 5 g Ca_3_(PO_4_)_2_, 5 g MgCl_2_·6H_2_O, 0.25 g MgSO_4_·7H_2_O, 0.2 g KCl and 0.1 g (NH_4_)_2_SO_4_ (Nautiyal 1999). All strains were inoculated by transfer from the 0.1 × TBA pH 5.0 media using inoculation loop and incubated for 6 weeks at 20 °C. The clearing zones around the colonies indicated phosphate solubilization by the isolates. The experiment was carried out in triplicate on separate plates.

### Siderophore production

Detection of siderophore production was carried out in chrome azurol S (CAS) agar plates. Removal of iron from the CAS dye by iron-chelating compounds results in a color change from blue to yellow/orange. The CAS medium was prepared according to the method described by Schwyn and Neilands (1987). Bacteria were collected from 0.1 × TSA plates, resuspended and washed twice with phosphate-buffered saline pH 6.5. An aliquot of 10 µL of bacterial suspension was spotted on CAS agar plates. Plates were checked daily for color change around each colony. *E. coli* WA321 was used as a positive control.

### *nif*H targeting PCR

The *nif*H gene targeting PCR was performed according to the modified protocol by Brankatschk et al. (2012). PCR amplification was performed in a 25-μL reaction mixture including DNA template, 0.6 μM of primers (*nif*HF/*nif*HR), 200 μM dNTPs, 1× of Taq buffer and 0.04 U FastStart High Fidelity *Taq* Enzyme Blend (Roche). The PCR were performed under the following conditions: initial denaturation step 5 min at 95 °C, followed by touchdown cycles of denaturation for 15 s at 95 °C, annealing starting at 63 °C with temperature decreases of 2 °C per two cycles and elongation at 72 °C for 45 s followed by 30 cycles with annealing at 53 °C. The final extension was extended to 10 min at 72 °C.

## Results and discussion

**Fig. 1:**
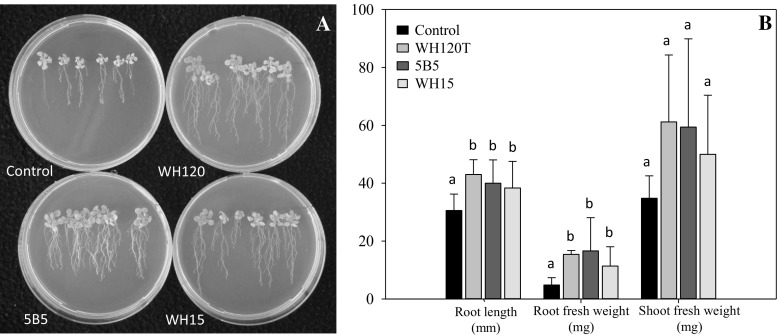
Effects of inoculation with *Acidobacteria* strains on *Arabidopsis thaliana* seedlings. Root tips of 5-day-old seedlings were inoculated by direct transfer with a loop of bacteria grown on 0.1 × TSA medium, pH 5.0. **A** Changes in morphology. Image was taken 3 weeks post-inoculation. **B** Changes in root length and fresh biomass. Different letters (*a*, *b*) indicate statistically significant differences (*P* < 0.05) between inoculated and control plants according to *t* test. *Error bars* represent SD (*n* = 6 plates each with six plants)

In this study, we tested three acidobacterial strains for possible interactions with *A. thaliana* (Col 0) roots. The growth of the plantlets was clearly positively affected by the presence of bacteria (Fig. 1). The presented results are shown with bacteria transferred directly from the solid media since this method of inoculation resulted in a stronger plant response. Root length, lateral root formation and root hair number were increased in plants exposed to *Acidobacteria* strains used in this study (Fig. 1A). Moreover, the root biomass increased significantly for plantlets inoculated with all three strains (Fig. 1B). The improved root architecture, more lateral branches and/or higher number of root hairs assist in more efficient water and nutrients uptake (Herder et al. 2010). Increased shoot biomass was also observed; however, the differences were not significant. We hypothesize that the stronger effect observed on plant growth with bacteria from the growth media in comparison with the bacterial suspension is not only related to higher bacterial biomass in the inoculum but also to the stress and longer adaptation time experienced by bacteria under the unfavorable culture conditions.

**Fig. 2:**
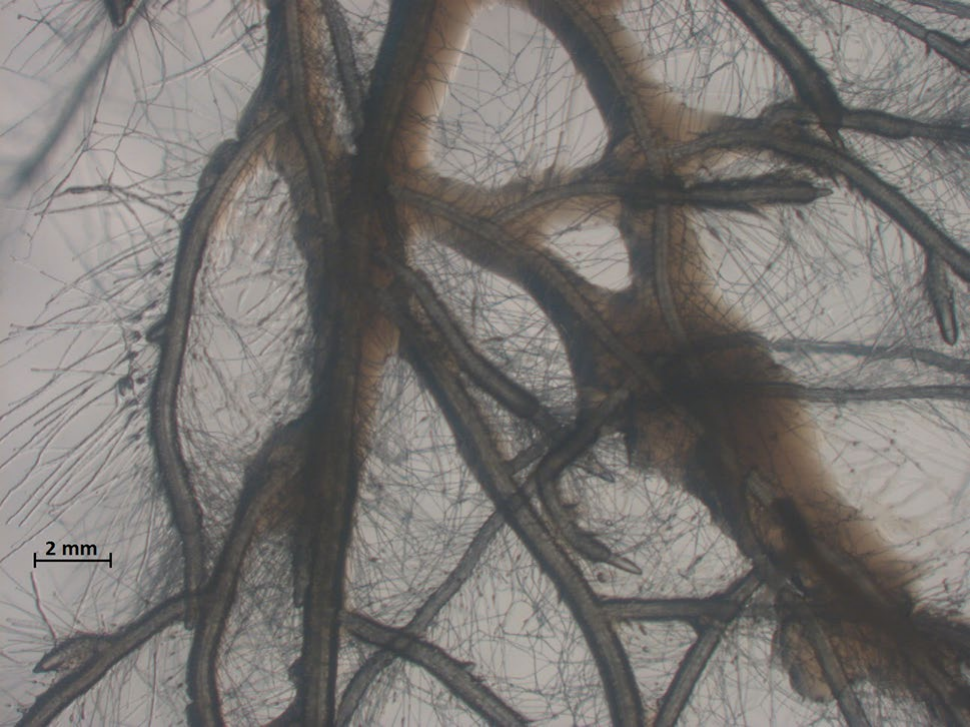
Root colonization and biofilm formation around *Arabidopsis thaliana* roots by strain 5B5

Bacterial adhesion, biofilm formation and growth along the root surfaces were observed for all three strains (Fig. 2). *Acidobacteria* strains *Granulicella* *paludicola*, *G. pectinivorans*, *G. aggregans* and *G. rosea* (Pankratov and Dedysh 2010), *Acidicapsa borealis* and *A. ligni* (Kulichevskaya et al. 2012) and *Terriglobus tenax* (Whang et al. 2014) were proven to produce extracellular polysaccharide. By genome mining, Ward et al. (2009) have suggested *Acidobacteria* being involved in soil matrix formation, water and nutrition trapping, or bacterial adhesion that lead to soil aggregate formation. However, here, for the first time we show that *Acidobacteria* strains produce exopolysaccharide (EPS) in the adhesion of bacteria to the root surfaces.

**Fig. 3:**
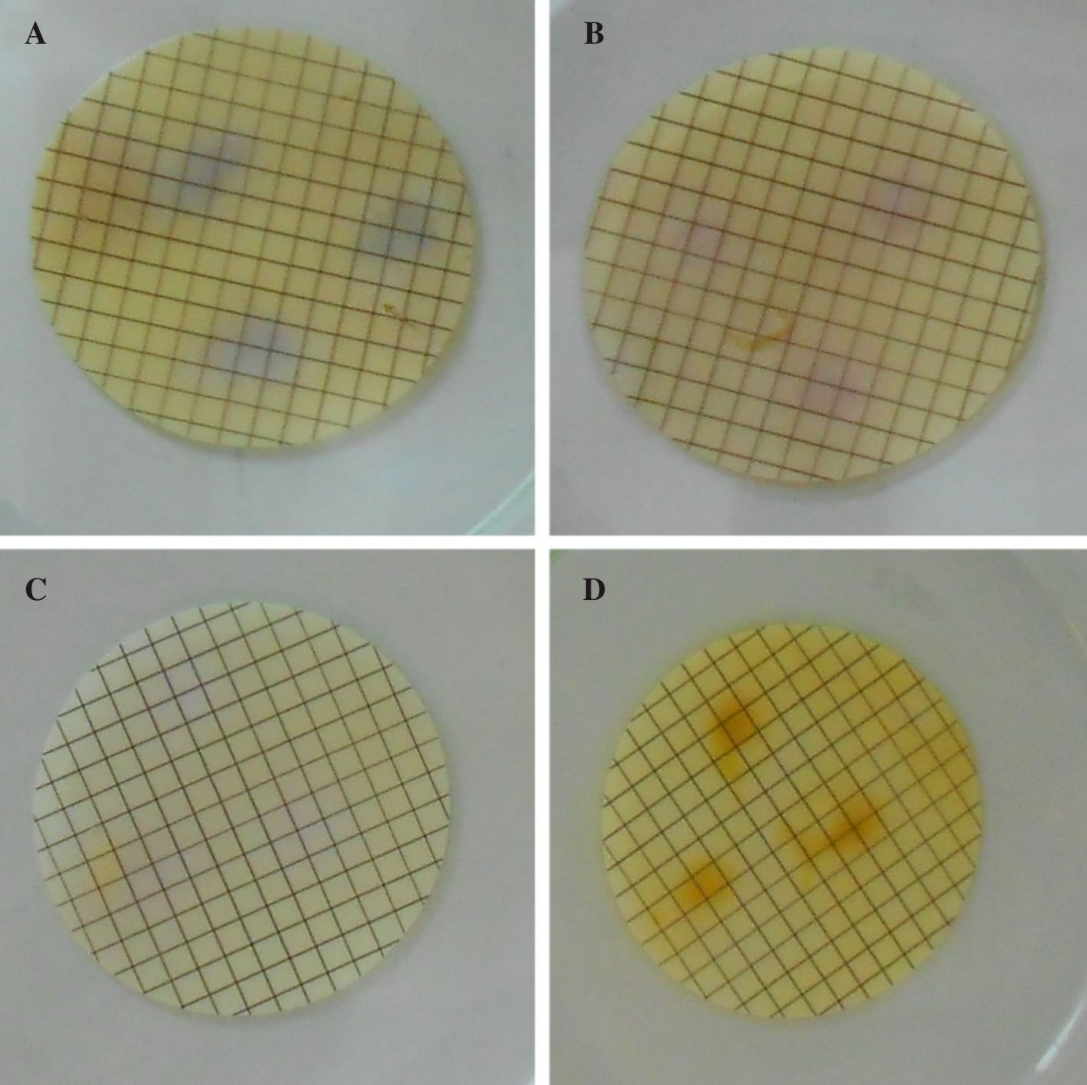
Isolates were tested for indole acetic acid (IAA) production by bacteria immobilization on a nitrocellulose membrane followed by washing the membrane with Salkowski reagent. *P. putida* IAC-RBal4 was used as a positive control. **A** *P. putida* IAC-RBal4, **B** strain WH120T, **C** strain 5B5 and **D** strain WH15

Bacteria can have positive effect on plant growth indirectly by acting as a biocontrol agent or directly by modulating plant hormone levels (Hayat et al. 2010) or/and by facilitating resource acquisition (mostly nitrogen, phosphorus and iron). Among the plant hormones, the auxin indole-3-acetic acid (IAA) has received most of the attention. As auxin production could best explain the observed changes in the plant phenotypes, we tested our strains for production of this phytohormone. A color change due to IAA production was observed for all three strains indicating all of them as positive for indolic substances production (Fig. 3).

**Fig. 4:**
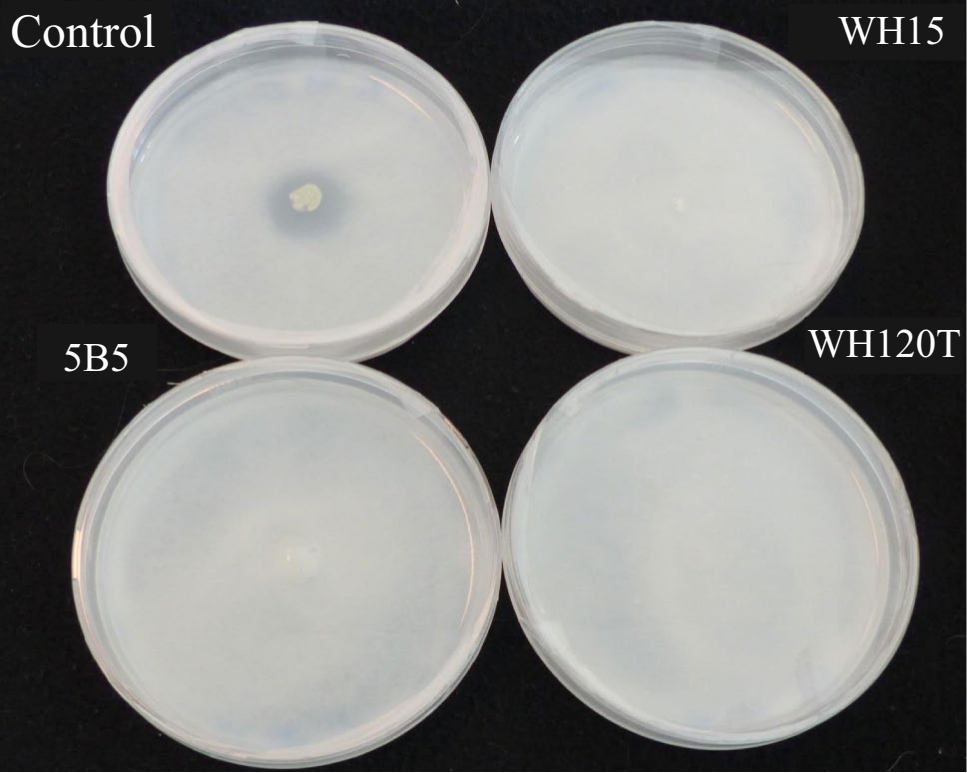
Isolates were tested for phosphate solubilization on agar plate using National Botanical Research Institute’s phosphate medium. *P. putida* IAC-RBal4 was used as a positive control

Moreover, we tested our strains for nutrient acquisition abilities. Phosphorus (P) is an important macronutrient for plant growth and development. However, in general, the concentration of soluble P in soil is quite low. It is postulated that bacteria can enhance the P acquisition of plants (Richardson and Simpson 2011). All three strains were proven to be not able to solubilize mineral phosphate (Fig. 4). Nevertheless, the enhanced P uptake by plants can be also achieved via hormonal stimulation of root growth, branching or root hair development mediated by IAA among others (Richardson and Simpson 2011).

**Fig. 5:**
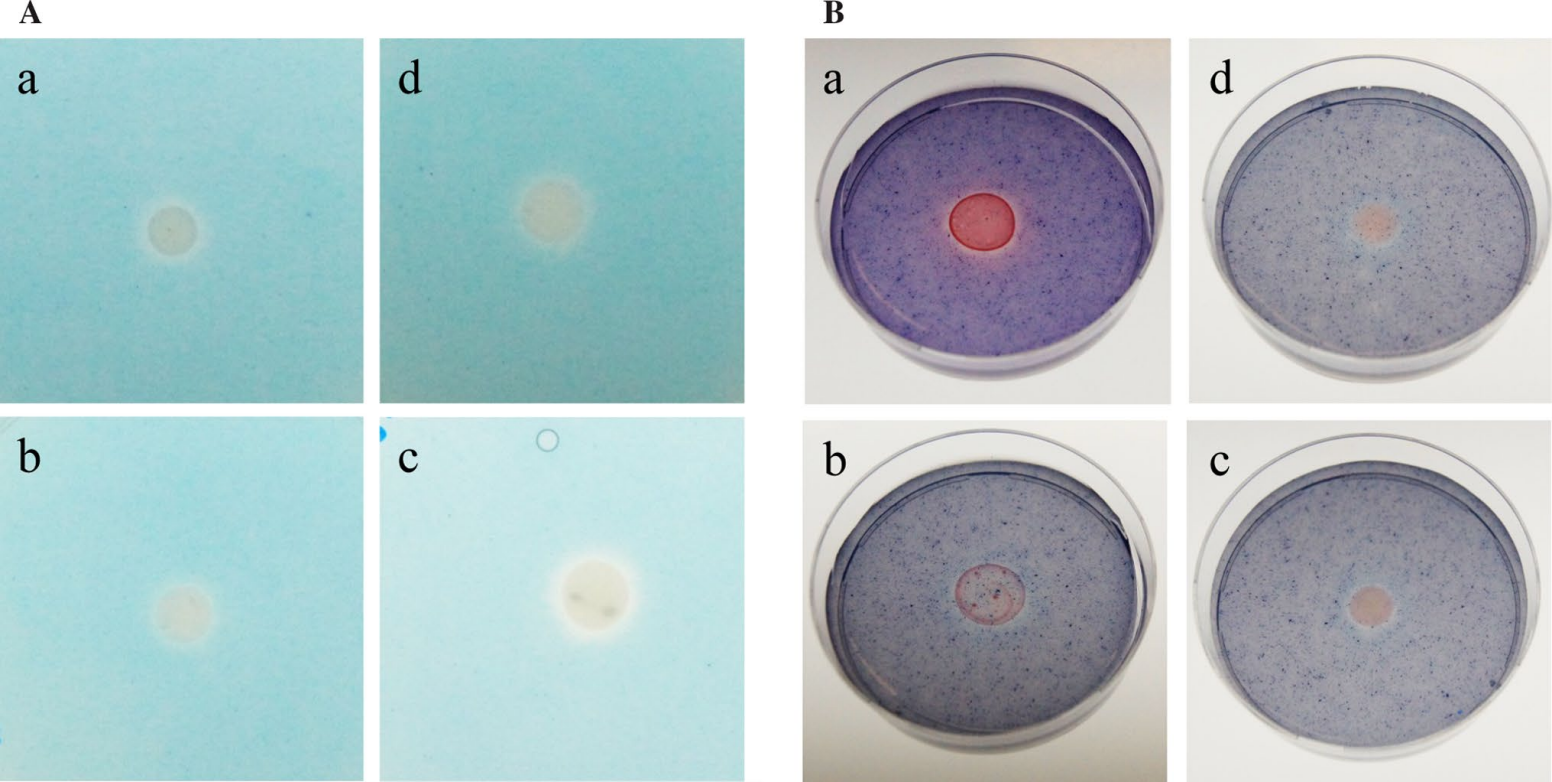
Isolates were tested for siderophore production on CAS medium. *E. coli* WA321 was used as a positive control. **A** CAS plates prepared with the MM9, pH 6.8. **B** CAS plates prepared with the MM9, pH 6.0 and supplemented with casamino acid. *a* *E. coli* WA321, *b * strain WH120T, *c* strain 5B5 and *d* strain WH15

On the other hand, the assay for siderophore production showed that all three strains react with the media (Fig. 5). Detection of siderophore production was carried out on chrome azurol S (CAS) agar plates. Removal of iron from the CAS dye by iron-chelating compounds results in a color change. The discoloration was observed for all strains with stronger discoloration zones observed around 5B5 and WH120T colonies than around colonies of strain WH15. Plants have high iron requirement but similarly to P, most of the iron in soil is in ferric form, which is unavailable for plant uptake (Hayat et al. 2010). The strategies of iron uptake by plants are similar to those from bacteria. Those include acidification of the rhizosphere resulting in reduction of Fe^3^–Fe^2^ or synthesis of Fe^3^ chelators (Morrissey and Guerinot 2009; Saha et al. 2013). Additional advantage of bacterial siderophore production is competition with pathogens by removing iron from the environment (Saha et al. 2013).

In order to test the ability of the strains to fix N_2_, we have carried out PCR targeting nitrogenase (*nif*H) gene. However, the results showed no evidence of such ability of tested strains. The absence of *nif*H is in agreement with acidobacterial genome mining studies (Ward et al. 2009). Up to now, there is no experimental evidence for the ability of *Acidobacteria* type strains to fix nitrogen.

## Conclusion

Based on our findings, we provide for the first time a direct evidence of active *Acidobacteria*–plant interaction and data indicating growth-promoting effects by *Acidobacteria*. We verified that a possible auxin production is involved in plant growth promotion. Although commonly used, the test conducted to unravel possible mechanisms of this phenomenon, for the first time was applied for *Acidobacteria*. Further studies are needed to better understanding the beneficial *Acidobacteria*–plant interaction as well as the mechanisms involved in such interaction. In addition, we conclude that EPS production during root colonization by *Acidobacteria* might be helpful in root adhering to soil particles and in root protection. Taking into account the dominance in abundance of this phylum in soil environment, the overall impact of *Acidobacteria* on plant growth may be significant and the results shown here indicate for the first time that *Acidobacteria* can act as plant growth-promoting bacteria.
